# The dynamics of Brazilian protozoology over the past century

**DOI:** 10.1590/0074-02760150386

**Published:** 2016-01

**Authors:** M Carolina Elias, Lucile M Floeter-Winter, Jesus P Mena-Chalco

**Affiliations:** 1 Instituto Butantan, Laboratório Especial de Ciclo Celular, São Paulo, SP, Brasil; 2 Instituto Butantan, Centro de Toxinas, Resposta-Imune e Sinalização Celular, São Paulo, SP, Brasil; 3 Universidade de São Paulo, Instituto de Biociências, Departamento de Fisiologia, São Paulo, SP, Brasil; 4 Universidade Federal do ABC, Centro de Matemática, Computação e Cognição, Santo André, SP, Brasil

**Keywords:** protozoology, pioneers, academic genealogy, scientific mapping method

## Abstract

Brazilian scientists have been contributing to the protozoology field for more than 100 years with important discoveries of new species such as*Trypanosoma cruzi* and *Leishmania* spp. In this work, we used a Brazilian thesis database (Coordination for the Improvement of Higher Education Personnel) covering the period from 1987-2011 to identify researchers who contributed substantially to protozoology. We selected 248 advisors by filtering to obtain researchers who supervised at least 10 theses. Based on a computational analysis of the thesis databases, we found students who were supervised by these scientists. A computational procedure was developed to determine the advisors’ scientific ancestors using the Lattes Platform. These analyses provided a list of 1,997 researchers who were inspected through Lattes CV examination and allowed the identification of the pioneers of Brazilian protozoology. Moreover, we investigated the areas in which researchers who earned PhDs in protozoology are now working. We found that 68.4% of them are still in protozoology, while 16.7% have migrated to other fields. We observed that support for protozoology by national or international agencies is clearly correlated with the increase of scientists in the field. Finally, we described the academic genealogy of Brazilian protozoology by formalising the “forest” of Brazilian scientists involved in the study of protozoa and their vectors over the past century.

The last decade of the XIX century is considered to be the period in which experimental protozoology began (Calkins 1911). At that time, protozoa were suspected of being the causative agents of only two human diseases: dysentery and malaria (Calkins 1911). Currently, protozoan parasites are recognised as the causative agents of some of most important human illnesses. For instance, amebiasis is the second leading cause of death due to parasitic diseases worldwide and causes approximately 40-100,000 deaths per year (Moraes et al. 2015). Approximately 6.5 million people are estimated to be infected with *Trypanosoma cruzi*, 1,300,000 new cases of leishmaniasis occur every year, and 214 million new cases of malaria have occurred in 2015 alone. Toxoplasmosis and giardiasis are also diseases caused by protozoa, both of which represent significant public health threats (who.int). These are examples of protozoa that are of medical interest and do not include protozoa of veterinary interest or free-living protozoa, which may be important environmental markers.

Therefore, protozoology has become a unique field of study, and an impressive amount of work has gone into detailing the biological aspects of this subject. Indeed, the study of protozoa has rapidly evolved from the molecular characterisation of eukaryotes through host-parasite interactions and ecoepidemiological aspects to therapeutic interventions. Several important mechanisms were first described in protozoa, such as nonconventional RNA polymerase II promoter sites (Clayton 2002), *trans*-splicing (Mayer & Floeter-Winter 2012) and RNA editing (Simpson et al. 2006). Some of these findings have been highly influential in other medical and biological fields, such as the discovery of telomeres protecting chromosomes in *Tetrahymena* (Blackburn & Gall 1978), the understanding of glycosylphosphatidylinositol protein anchor structures (Ferguson et al. 1985, Ferguson 1999), and the delineation of the respective roles of the T-helper (Th)1 and Th2 lymphocyte subsets against infectious agents in studies using *Leishmania major*-infected mice (Heinzel et al. 1989).

In Brazil, where some protozoan infections are endemic, these aetiological agents and their vectors have attracted considerable interest from researchers and students. A few “founding fathers” (pioneers) of this field of science have nurtured later generations of protozoologists, resulting in the development of a solid network of 100 years of scientists.

The evolution of science is the pillar that provides a solid foundation for the development of society by creating the means to face the challenges ahead (Cordova et al. 2015). The study of the origin of a scientific area and the identification of the motives behind its development in new disciplines provide important contributions to the understanding of future needs. This academic genealogy allows the development of qualifying studies based on the training of new researchers.

Academic genealogy was defined by Sugimoto (2014)as a quantitative study of the intellectual heritage perpetuated through academic orientation relationships among professors (i.e., mentors or supervisors) and their students. Various questions can be answered by building an academic genealogy. Sugimoto (2014) proposed five types of academic genealogies: honorific, egotistical, historical, paradigmatic, and analytical. These categories are not mutually exclusive, and most academic genealogies can be classified into at least two of these types.

In this work, we followed the development of protozoology in Brazil using a historical and paradigmatic approach. Systematic data collection was performed through the analysis of formal thesis orientation and was organised to create a chain of mentorships, resulting in the construction of our academic genealogy.

## MATERIALS AND METHODS

**Fig. 1:**
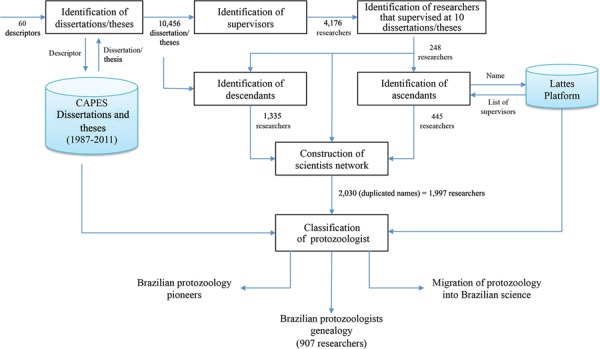
: diagram of the tracking of Brazilian protozoologists. Each block represents a process and each arrow represents the information flow between processes.

In this work, the identification of researchers associated with protozoology who influenced (supervised) other researchers over a 100 year timespan was performed through seven processes (Fig. 1). The methodology was based on the analysis of two sources of Brazilian academic information that allowed the tracing of scholarly interactions among researchers.

*Process 1 - Identification of dissertations and theses related to protozoology -* A local repository extracted from the Coordination for the Improvement of Higher Education Personnel (CAPES) thesis database (Mena-Chalco & Rocha 2014) was used in this process. We identified 10,456 dissertations or theses with at least one descriptor related to protozoology (Supplementary Table I). This quantity represented 1.7% of the 607,389 dissertations or theses registered in the local repository.

*Process 2 - Identification of supervisors of Masters and PhD students in protozoology* - In this process, we extracted all supervisors’ names from the dissertations/theses identified in process 1. The process handled incomplete or similar names using approximate string matching. Two names were considered the same/similar if the Levenshtein distance (Levenshtein 1966) between them was equal to 2. This process allowed us to identify 4,176 researchers related to the protozoology field.

*Process 3 - Identification of representative protozoologists in terms of the quantity of supervisions registered in the CAPES thesis database* - In this process, researchers who supervised at least 10 projects were selected to generate a list of 248 supervisors (6% of 4,176 researchers). This threshold was considered suitable for manual inspection. Supplementary Table II presents the complete list of names obtained through this process.

*Process 4 - Identification of ancestors of the representative protozoologists* - This process was performed recursively for each supervisor identified in the Lattes Platform. First, the name of the supervisor was identified and associated with its Lattes CV. Then, the same approach was undertaken for the supervisors of the supervisors until the inability to identify a new supervisor was reached. We identified 445 researchers as the ancestors of the 248 representative protozoologists.

*Process 5 - Identification of the descendants of the representative protozoologists* - This process was accomplished by selecting the PhD students (process 1) who were supervised by the researchers obtained in process 3. We identified 1,335 direct descendants from the group of 248 representative protozoologists.

*Process 6 - Construction of a scientist network -* In this process, the researchers identified in processes 3, 4 and 5 were used to generate a supervisor network (i.e., a directed graph where each node represented a researcher, and the edge represented the relationship between 2 researchers). Duplicated names were processed manually. A list of 1,997 complete names was generated as the result of this process (Supplementary Table III).

*Process 7 - Classification of protozoologists -* This process was performed by manual inspection of each researcher identified in the previous process. Information from the protozoology field was associated with each of the 1,997 researchers using the Lattes Platform and academic repositories.

## RESULTS

**Fig. 2:**
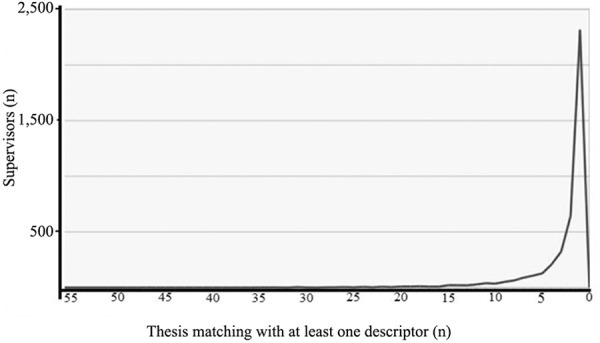
: relationship between the number of supervisors and number of theses generated from 1987-2011 containing at least one descriptor. Supplementary Table I containing 60 descriptors was used to screen the Coordination for the Improvement of Higher Education Personnel database. The graph indicates how many scientists supervised a different number of theses containing at least one descriptor as a keyword.

*Identification of scientists actively working in protozoology* - This work aims to understand the past and present of Brazilian protozoology and the migration of scientists from protozoology to other fields and from other fields to protozoology. Academic dissertations and theses are an important source of information concerning the growth and evolution of science (Andersen & Hammarfelt 2011). Therefore, we accessed a data collection containing most of the Brazilian protozoologists from the CAPES database combined with the Lattes examination. By searching for the supervisors of actively working protozoologists, the supervisors of these supervisors, and so on, we identified the pioneers of this field in Brazil. Similarly, by searching for students trained by these actively working protozoologists, we identified other people currently working in the field. To establish the names of actively working protozoologists to nucleate our search, we prepared a set of 60 words (descriptors) (Supplementary Table I) for use as keywords to screen theses (Masters or PhD) present in the local CAPES thesis database, which contained all theses completed in Brazil from 1987-2011 (Mena-Chalco & Rocha 2014). A total of 10,456 Masters or PhD theses contained at least one such descriptor. This number corresponded to 1.7% of the total theses presented in the same period throughout all fields. A total of 4,176 researchers supervised these 10,456 dissertations/theses. As expected, the number of scientists diminished when we increased the number of works supervised per researcher (Fig. 2). For operational reasons, it might not be possible to analyse in detail all the 4,176 researchers (advisors) and the relevant factors considered in the adopted methodology. Therefore, only researchers with at least 10 dissertations/theses supervised in the field of protozoology were considered in our study (248 scientists) (Supplementary Table II). With a threshold of five or two dissertations, for instance, the number of researchers increases by 276% (689 researchers) or 745% (1,864 researchers), respectively. With the threshold adopted, important pioneers in the field of protozoology are included in the analyses, but we recognise that this empirical value might penalise early career researchers. However, we believe that this arbitrarily selected threshold of 10 dissertations/theses was appropriate to define a selected group of representative researchers. A total of 36% of these researchers are or were members of the Brazilian Society of Protozoology in the period between 2000-2015.

**Fig. 3:**
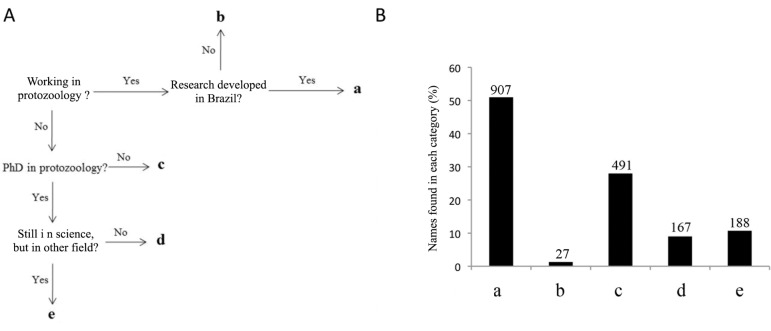
: classification of scientists according to Lattes CV. A: pathway followed during inspection with Lattes CV; B: frequency of each category described in A; a: scientists working or who worked in the past in protozoology in Brazil; b: scientists working in protozoology outside of Brazil that were advisors of Brazilians; c: scientists that did not work in protozoology during their PhD or establish a research interest in this field but were advisors of scientists that migrated to protozoology; d: persons who developed their PhD in protozoology but are now involved in activities other than science; e: scientists who developed their PhD in protozoology but established research in another field.

*Identification of Brazilian protozoologists* - We used the complete list of names in Supplementary Table II as a starting point to search for the people supervised by these people and *vice versa* (i.e., scientists who supervised these people). Using automated analysis of the local CAPES thesis database, we found students whom these researchers supervised (descendants). We did not search for students of these students because some of them were still settling, which could compromise our analysis. Based on the automated analysis of their Lattes CVs, we could also follow the advisors’ ancestors by searching for their supervisors and the supervisors of these supervisors available on the Lattes CV database (Fig. 1). As shown below, the analyses also allowed the identification of Brazilian supervisors who worked outside of protozoology and supervisors that were from other countries; thus, we could identify the seeds of Brazilian protozoology. These analyses provided a list of 1,997 names that were manually inspected through the Lattes CV examination, following the pathway shown inFig. 3A. We used the following criteria to establish that a researcher is/was working in the field of protozoology. Researchers studying the biology of protozoa or protozoa-host interaction were considered protozoologists. Additionally, scientists investigating protozoa vectors were considered protozoologists. In contrast, researchers working with protozoa but investigating the clinical aspects of diseases (e.g., mostly ophthalmologists, dermatologists, and cardiologists who study diseases caused by protozoa) were not considered protozoologists. From the total of 1,997 names found, some were not included in the Lattes database. This discrepancy might have occurred for four different reasons: (i) misspelling of names, (ii) people who are no longer in science, (iii) foreign supervisors, and (iv) people who worked in protozoology before the Lattes CV was created. To include this last group in our analysis, we searched for these people in the CAPES database or in public repositories to identify their ancestors and the field in which they worked. To include supervisors from other countries, we searched for these people in PubMed to verify the field in which they work/worked. Finally, 10.86% of the 1,997 names could not be found in either the Lattes Platform, CAPES database, or public repositories and were removed from the forward analyses. These analyses allowed the classification of researchers into five different categories: (a) scientists who are working or previously worked in protozoology in Brazil, (b) scientists working in protozoology outside of Brazil who were advisors of Brazilians, (c) scientists who did not work in protozoology during their PhD and did not establish a research interest in this field but were advisors of scientists who migrated to protozoology, (d) people who obtained their PhD in protozoology but were now involved in activities other than science, and (e) scientists who developed their PhD in protozoology but established a research interest in another field (Supplementary Table III). The frequency of each group is presented in Fig. 3B. Based on the data, 907 names were identified as Brazilian protozoologists (classified as group a).

**Fig. 4:**
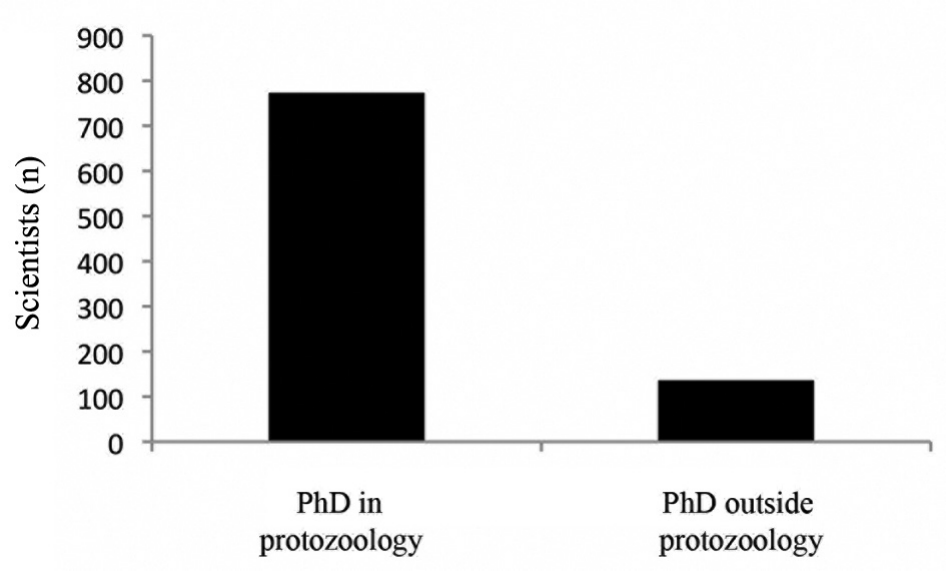
: analysis of fields where scientists classified as developed their PhD. Scientists classified in category “PhD in protozoology” were divided into those who developed and those who did not develop PhDs in protozoology.

**Fig. 5:**
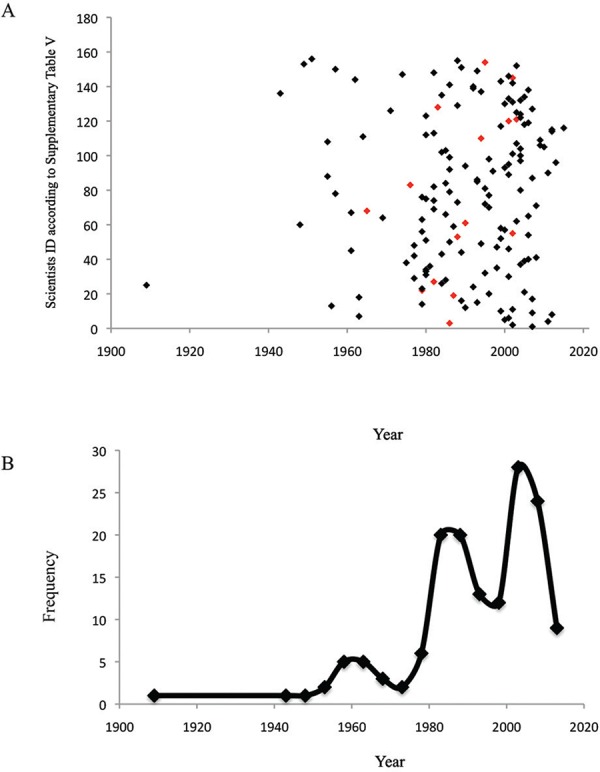
: incoming scientists into protozoology. A: the graph shows the year in which each researcher who did not develop a PhD in protozoology published their first paper in this field. Red dots indicate the year that scientists who developed a PhD in protozoology outside of Brazil published their first paper in Brazil after their PhD; B: frequency of scientists immigrating into Brazilian protozoology according to the year of arrival.

*Identification of Brazilian protozoology pioneers* - To identify researchers who were Brazilian protozoology pioneers, we first assumed that people who were awarded a PhD in protozoology in Brazil were not pioneers but that the pioneers would be their advisors. Therefore, we concluded that the pioneers were among the scientists who were awarded PhD degrees in other fields and then migrated to protozoology or people who were awarded PhDs in protozoology outside Brazil and then came to our country to establish a group here. Among the researchers classified as group a (Brazilian protozoologists), we manually searched the Lattes CV and the CAPES database for ones who were (766) and were not (141) awarded a PhD in protozoology (Fig. 4, Supplementary Table IV). Additionally, we searched the Lattes CV for researchers who were awarded a PhD in protozoology outside Brazil (15) (Supplementary Table V, in bold). Then, we determined in what year these researchers who did not receive a PhD in protozoology (141 names) or who received a PhD outside Brazil (15 names; a total of 156 names) established a protozoology group in Brazil. For scientists who arrived from other fields, we searched the Lattes CV for the year of their first paper published in protozoology (Supplementary Table V). For scientists who received their PhD outside of Brazil, we searched the Lattes CV for the year in which they published their first paper after their PhD from a position in Brazil (Supplementary Table V, in bold). Carlos Chagas’s paper describing *T. cruzi* was not found in PubMed but was considered due to its relevancy. Using this date as the year the scientist entered Brazilian protozoology, we plotted a graph of this incoming year for each of the 156 scientists who arrived in protozoology (Fig. 5A). Additionally, we plotted a graph showing the frequency of this influx according to the year of migration (Fig. 5B). From both analyses, it was clear that there were three waves of immigration into Brazilian protozoology: (i) up to and including 1974, (ii) between 1978-1993, and (iii) between 1998-2013. However, the drop observed after 2013 might be artificial because the collection of data is recent. Thus, we concluded that the pioneers (i.e., founders or precursors) of Brazilian protozoology were the 20 scientists who migrated into the field up to 1974: Carlos Chagas, Samuel Pessoa, Hertha Meyer, Zigman Brener, Wladimir Lobato Paraense, Leonidas Deane, Maria von Paumgartten Deane, Amilcar Viana Martins, José Rodrigues da Silva, Washington Luiz Tafuri, Erney Felicio Plessmann de Camargo, Jayme Neves, Aluízio Prata, Thales de Brito, Astolpho Ferraz de Siqueira, Jeffrey Jon Shaw, Mario Endsfeldez Camargo, Isaac Roitman, Raymundo Martins de Castro, and Walter Colli.

*Migration of protozoology into Brazilian science* - We also investigated the immigration and emigration of scientists to and from Brazilian protozoology. A total of 17.1% of the studied researchers immigrated into protozoology, based on the number of people who entered protozoology from other fields and arrived from protozoology outside of Brazil (156 names/907 total). Researchers who stayed in protozoology after their PhD corresponded to 68.4% of individuals, based on the 770 protozoologists who received a PhD in protozoology and were still acting as protozoologists, those who received a PhD in protozoology and were involved in other activities but still involved in science [group d: 167 (Supplementary Table III)], and those who received a PhD in protozoology and established a group in another field [group e: 188 (Supplementary Table III)]. Finally, we determined the percentage of people who left Brazilian protozoology to contribute to other fields of Brazilian science. To obtain this number, we divided the number of people classified as group e in Supplementary Table III (188) by the total number who received a PhD in protozoology. This analysis demonstrated that protozoology provided 16.7% of its PhDs to other fields of science.

**Fig. 6:**
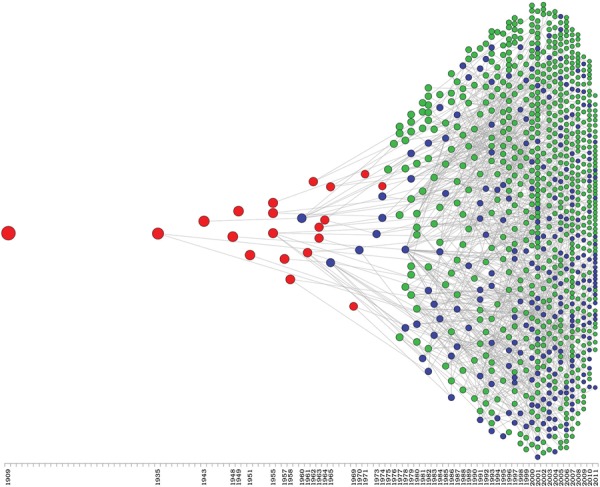
: academic genealogy of Brazilian protozoology protozoologists (group a in Fig. 3) were included in this graph. Each circle represents one researcher and lines represent student-supervisor relationships. Persons identified as pioneers are shown in red, persons directly influenced by pioneers (students or students of their students) are shown in blue, and persons who arrived in protozoology after the pioneers and their descendants are shown in green. The x-axis shows the year of the conclusion of the PhD in protozoology or, for those who did not receive a PhD in protozoology, the year that they migrated to protozoology.

*Academic genealogy of Brazilian protozoologists* - Finally, we constructed an academic genealogy (Fig. 6) that included all names classified as protozoologists to reflect the scenario of protozoology in Brazil. The resulting structure is a forest containing one tree for each scientist who entered Brazilian protozoology. A detailed view of this academic genealogy is available from professor.ufabc.edu.br/~jesus.mena/brazilian-protozoology-scenario/. It is clear that protozoology expanded in the mid-1970s. It is also clear that protozoology today consists of the descendants of the pioneers as well as other scientists who migrated to protozoology and supervised their students in this field.

## DISCUSSION

The analysis of the dynamics of Brazilian protozoology over the past century based on database searches from 1987-2011 allowed us to track and identify scientists who made important contributions by acting as the pioneers of this field. Moreover, we could determine the percentage of scientists immigrating to protozoology as well as the percentage of scientists who were supervised in protozoology but established a research group in another field. The field of Brazilian protozoology comprises at least 907 researchers. Certainly, there are other protozoologists who were not covered by our criteria and therefore were not included in our analysis. The interesting result is that a large number of people working in this field are the result of the efforts of 20 pioneers who introduced protozoa as biological models or drug targets in the period between 1909-1974. In a seminal paper published in*Memórias do Instituto Oswaldo Cruz*, Carlos Chagas described*T. cruzi* and thereby introduced the study of protozoology in Brazil (Chagas 1909). Surprisingly, no descendants were identified for Carlos Chagas, probably because his descendants were clinical doctors and therefore were not classified as protozoologists according to the criteria used in this study. However, Carlos Chagas’s work at the beginning of the previous century was so complete in showing the causal agent of a disease and its relationship to an insect vector that it still inspires researchers today.

In the 1940s, Samuel Pessoa inaugurated his huge contribution to the field by studying the behaviour of *Leishmania* in tissues (Pessoa & Barreto 1945) and the geographic distribution of phlebotomines (Barreto & Pessoa 1946). Hertha Meyer published on *T. cruzi* cultivation (Meyer & de Oliveira 1948); this field of study evolved to include her contribution regarding the structural analysis of this organism. In the 1950s, the study of protozoa in Brazil expanded with Amilcar Viana Martins, Leonidas Deane, and Maria Deane, who investigated the epidemiology of leishmaniasis (Deane & Deane 1954, Martins et al. 1956), Zigman Brener and Washington Luiz Tafuri, who contributed to our understanding of Chagas disease (Brener 1952, de Queiroz & Tafuri 1957), José Rodrigues da Silva, who investigated hepatic problems related to *Leishmania* and amoeba infections (da Silva & de Paola 1957, daSilva & Torres 1957), and Wladimir Lobato Paraense, who was interested in *Plasmodium* (Paraense 1952). Then, over the next two decades, a new group of scientists migrated into protozoology. Their studies included the detection and treatment of *Trypanosoma*,*Leishmania*, *Toxoplasma*, and*Plasmodium* by Raymundo Martins de Castro (Sampaio et al. 1971), Aluízio Prata (Prata 1963), Mario Camargo (Camargo 1964b), and Thales de Brito (de Brito et al. 1962), the biology of trypanosomatids by Walter Colli (Alves & Colli 1974), Erney Camargo (Camargo 1964a), and Isaac Roitman (Roitman 1969), and epidemiological approaches to studying diseases and hosts by Jeffrey Shaw (Shaw & Lainson 1968), Jayme Neves (Neves et al. 1961), and Astolpho Ferraz de Siqueira (Barreto et al. 1963). These studies concluded the first phase of Brazilian protozoology. The co-authors of the works cited above certainly contributed to Brazilian protozoology and could be identified as founders of Brazilian protozoology.

The pioneers/founders, together with the students they supervised, nucleated the Brazilian protozoologist network. Thus, the environment favouring the construction of this field in Brazilian science was created. By supervising new students, organising scientific meetings and working side by side with agencies to create programmes for financial support, these scientists solidified the foundations of Brazilian protozoology and allowed the influx of new scientists into this area.

Immigration occurred in two waves. The entry of scientists between 1978-1993 might be a consequence of the creation of the Integrated Program for Endemic Diseases (PIDE), which was the Funding Programme from the Brazilian Council for Science Development (CNPq) that operated between 1976-1986. This programme invested the equivalent of 12 million American dollars in groups working in approximately 200 projects in endemic diseases (Gonçalves et al. 1988). Due to its differentiated financial policy, which means an initiative to influence the development of a special area, the programme had a huge impact on attracting more groups to work in protozoology, and our data reinforce this importance.

The second wave of incoming scientists occurred after 1998 and might be the consequence of the elevated number of fellowships and resources offered by the CNPq. In this sense, national politics favoured Brazilian science, and protozoology took advantage of this situation. However, we cannot forget the contribution of International Funding Programmes such as the Tropical Diseases Research (TDR) of the World Health Organization.

By tracking students who completed their PhD in protozoology, we observed that 85% of them were still involved with science. Considering that the other 15% might include people who teach in private universities using the knowledge acquired during their PhD, we can conclude that the resources invested in protozoology were very well returned to society. However, it is time to reflect on whether the current number of students completing their PhDs will be harnessed in Brazil as has occurred in the past.

The scenario of protozoology in Brazil, as shown in Fig. 6, presents an increasing trend. Although the immigration dynamics presented in Fig. 5 showed a possible decrease in interest, Brazilian protozoology is the result of the work of pioneers and also (as evidenced by the green dots in Fig. 6) the consequence of the immigration of many scientists to protozoology who supervised their students in this area. It is important to reinforce that this flux requires financial support or strong funding programmes such as the PIDE and TDR.

Our goal in this communication was to show the dynamics of protozoologists and the impact of protozoology on Brazilian science. The same approach can be used to study contributions in other fields. The use of different criteria can group people in various ways to reveal other trees and identify other pioneers, even in protozoology. The genealogy presented here is one of multiple possible methods to track our past and hopefully point to our future.

From Isaac Newton to Stephen Hawking, the idea of “having seen further by standing on the shoulders of giants” has been used in science to recognise past mentors for new discoveries. We expect that in addition to serving as a source for research on the historical and paradigmatic aspects of Brazilian protozoology and positioning the contributions of the field to Brazilian science, this paper may be seen as a form of acknowledgement of the pioneering researchers who built the foundations of our work and inspired new generations of protozoology.

## Figures and Tables

**TABLE I:**
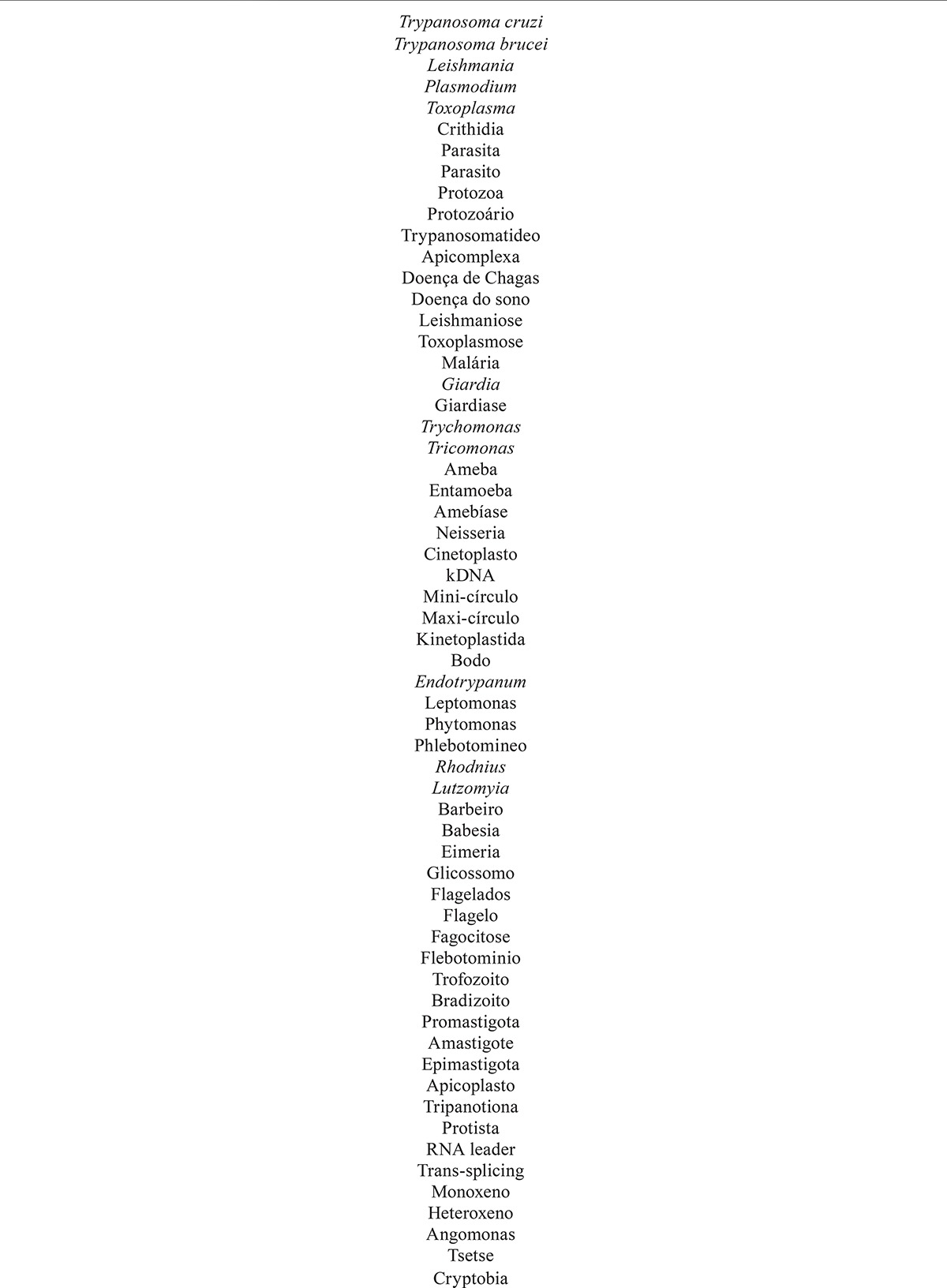
Descriptors used to screen theses on Coordination for the Improvement of Higher Education Personnel database

**TABLE II:**
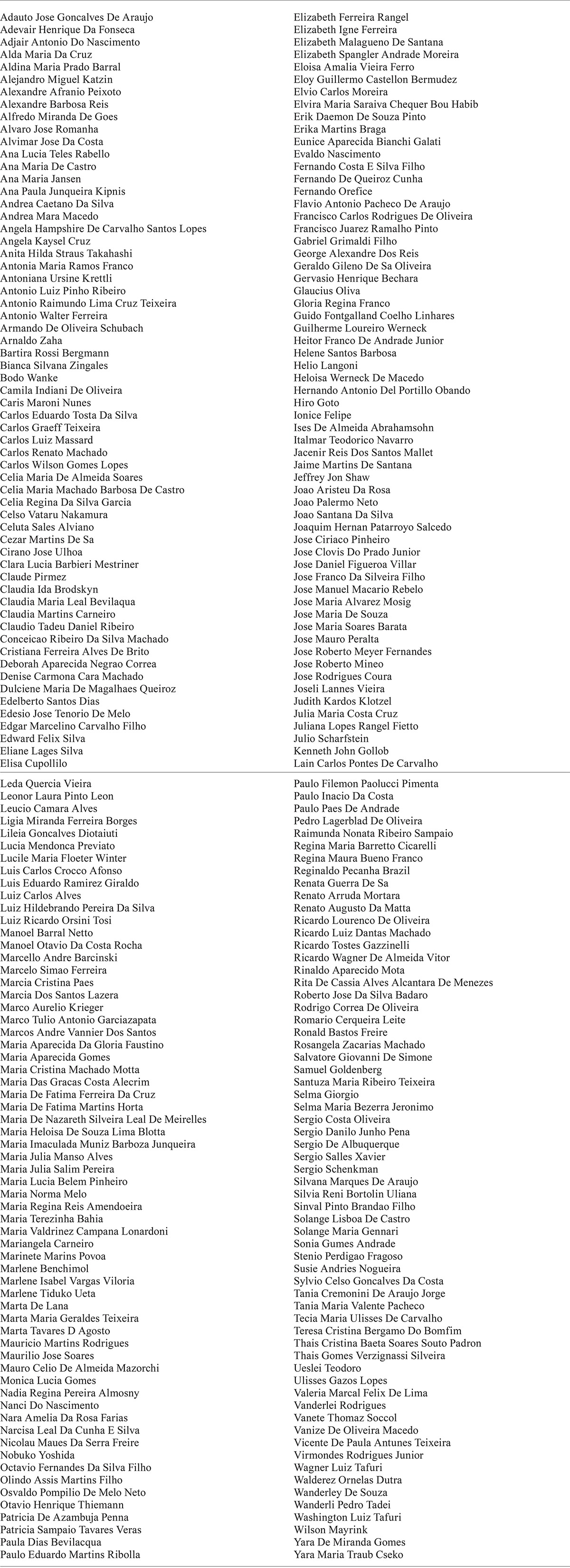
Scientists that supervised at least 10 theses/dissertations containing at least one of the 60 descriptors present on Supplementary Table I

source: Coordination for the Improvement of Higher Education Personnel database in the period from 1987-2011.

**TABLE III:**
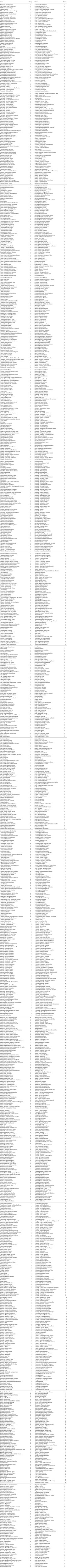
Classification of names according to diagram in Fig. 3A

a: scientists working or who worked in the past in protozoology in Brazil; b: scientists working in protozoology outside of Brazil that were advisors of Brazilians; c: scientists that did not work in protozoology during their PhD or establish a research interest in this field but were advisors of scientists that migrated to protozoology; d: persons who developed their PhD in protozoology but are now involved in activities other than science; e: scientists who developed their PhD in protozoology but established research in another field.

**TABLE IV:**
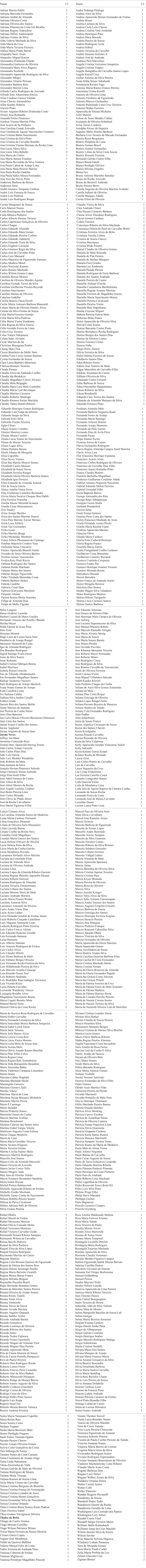
Classification of names that developed (1) or not (2) PhD in protozoology

bolded names: persons who developed PhD in protozoology having as supervisor a nonprotozoologist.

**TABLE V:**
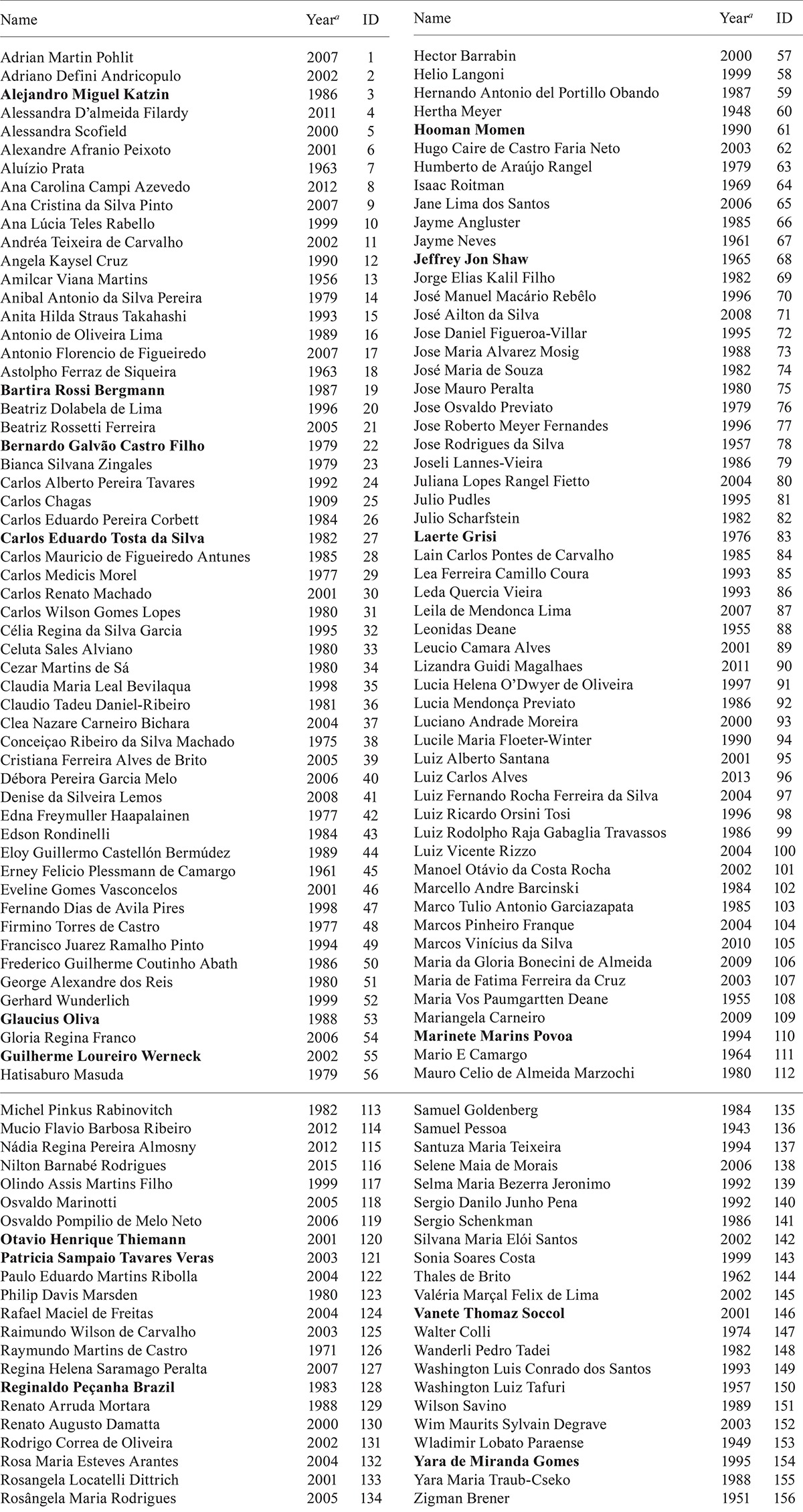
Year that persons who did not develop PhD in protozoology published in this field

*a*: the year that scientist published his first paper in Brazil after his PhD; bolded names: persons who did develop PhD in protozoology outside Brazil.
